# Advantages of Endoscopically Assisted Surgery for Attic Cholesteatoma

**DOI:** 10.1155/DTE.7.99

**Published:** 2001

**Authors:** Kazuhiro Aoki

**Affiliations:** Department of Otolaryngology JIKEI University School of Medicine 3-25-8 Nishishinbashi, Minato-ku Tokyo 105-8461 Japan

## Abstract

Combined use of an operating microscope and a middle ear endoscope seems to be helpful for selecting an appropriate surgical technique and for identifying more patients in whom cholesteatoma can be removed by a trans-canal approach alone. To investigate whether attic cholesteatoma can be treated by a trans-canal approach alone, a review was performed of patients who had undergone endoscopically assisted tympanoplasty and the outcome of surgery was compared with the preoperative CT findings. Using a rigid endoscope (3 mm in diameter and 6 cm in length with a viewing angle of 30°), twenty eight patients were examined to determine whether total resection of the cholesteatoma was possible by trans-canal atticotomy alone. According to the CT findings, total resection of cholesteatoma was possible by trans-canal atticotomy combined with the use of a rigid endoscope not only in 4 patients with the shadow localized in the epitympanum on preoperative CT scans but also in 18 out of 24 patients with the shadow extending from the epitympanum to the distal mastoid air cells. This study indicates that the trans-canal approach with endoscopic guidance is a useful technique for the treatment of cholesteatoma.


*Diagnostic and Therapeutic Endoscopy*, Vol. 7, pp. 99–107
Reprints available directly from the publisher
Photocopying permitted by license only

© 2001 OPA (Overseas Publishers Association) N.V.
Published by license under
the Harwood Academic Publishers imprint,
part of Gordon and Breach Publishing
a member of the Taylor & Francis Group.
All rights reserved.

### Advantages of Endoscopically Assisted Surgery for Attic Cholesteatoma

KAZUHIRO AOKI*

*Department of Otolaryngology, JIKEI University School of Medicine, 3-25-8, Nishishinbashi, Minato-ku, Tokyo 105-8461, Japan*

*(Received 14 June 2001; Revised 27 August 2001; In final form 27 August 2001)*

Combined use of an operating microscope and a middle ear endoscope seems to be helpful for selecting an appropriate surgical technique and for identifying more patients in whom cholesteatoma can be removed by a trans-canal approach alone. To investigate whether attic cholesteatoma can be treated by a trans-canal approach alone, a review was performed of patients who had undergone endoscopically assisted tympanoplasty and the outcome of surgery was compared with the preoperative CT findings. Using a rigid endoscope (3 mm in diameter and 6 cm in length with a viewing angle of 30°), twenty eight patients were examined to determine whether total resection of the cholesteatoma was possible by trans-canal atticotomy alone. According to the CT findings, total resection of cholesteatoma was possible by trans-canal atticotomy combined with the use of a rigid endoscope not only in 4 patients with the shadow localized in the epitympanum on preoperative CT scans but also in 18 out of 24 patients with the shadow extending from the epitympanum to the distal mastoid air cells. This study indicates that the trans-canal approach with endoscopic guidance is a useful technique for the treatment of cholesteatoma.

*Keywords*: Attic cholesteatoma; Direct vision; Endoscopic ear surgery; Rigid endoscope; Trans-canal approach

### INTRODUCTION

When deciding on the treatment policy for an attic cholesteatoma, whether the cholesteatoma can be treated by atticotomy alone or whether the mastoid antrum must be opened is generally determined according to the depth of invasion based on preoperative examination of the tympanic membrane and the results of CT scanning. However, examination of the tympanic membrane alone often fails to provide a clear distinction of an appropriate approach between the atticotomy alone and the combined approach with mastoidectomy, and the cholesteatoma may also be masked by other lesions such as granulations on CT scans. Therefore, the final strategy for surgical treatment is often decided based on the operative findings. At that time, combined use of an operating microscope and a middle ear endoscope seems to be

*Tel.: +81-3-3433-1111. Fax: +81-3-3435-8463. E-mail: aoki@jikei.ac.jp helpful for selecting an appropriate surgical technique and for identifying more patients in whom cholesteatomas can be removed by a trans-canal approach alone. To investigate whether attic cholesteatomas can be treated by a trans-canal approach alone, i.e. atticotomy, a review was performed of patients who had undergone endoscopically assisted tympanoplasty and the outcome of surgery was compared with the preoperative CT findings.

### MATERIALS AND METHODS

Prior to surgery, CT scans were obtained in the axial, sagittal, and coronal planes in 28 patients with an attic cholesteatoma who were scheduled for endoscopically assisted tympanoplasty over the past one year of 1999. CT scanning was done to ascertain whether the lesion was localized to the epitympanum or was spreading beyond the mastoid antrum and into the air cells of the mastoid process. Using a rigid endoscope (3 mm in diameter and 6 cm in length with a viewing angle of 30°), all patients were examined to determine whether total resection of the cholesteatoma was possible by trans-canal atticotomy alone (Fig. 1).

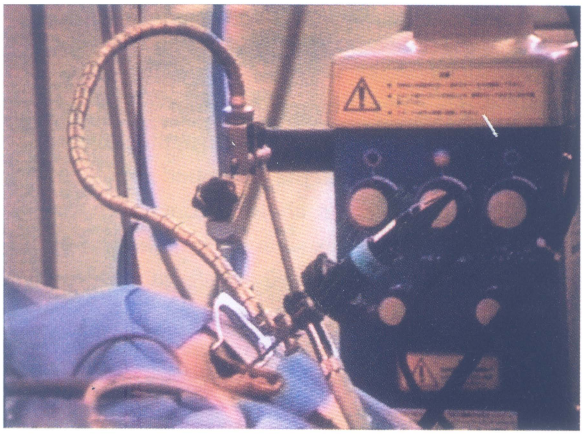

FIGURE 1 Combined use of an operating microscope and a middle ear endoscope.

TABLE I CT, operative findings and outcome of surgery

| CT findings (shadow) | Operative findings (cholesteatoma expansion) | | Outcome of surgery (with or without mastoidectomy) |
|---|---|---|---|
| Group A (3 cases) | Lateral of ossicles 3 cases | | Without mastoidectomy Trans-canal approach alone |
| Group B (1 case) | Epitympanum 1 case | | |
| Group C (24 cases) | Lateral of ossicles 2 cases<br>Epitympanum 3 cases | } With granulation and fluid 5 cases | Without mastoidectomy Trans-canal approach alone |
| | Epitympanum 13 cases | With granulation 13 cases<br>Mastoid antrum 6 cases | With mastoidectomy |

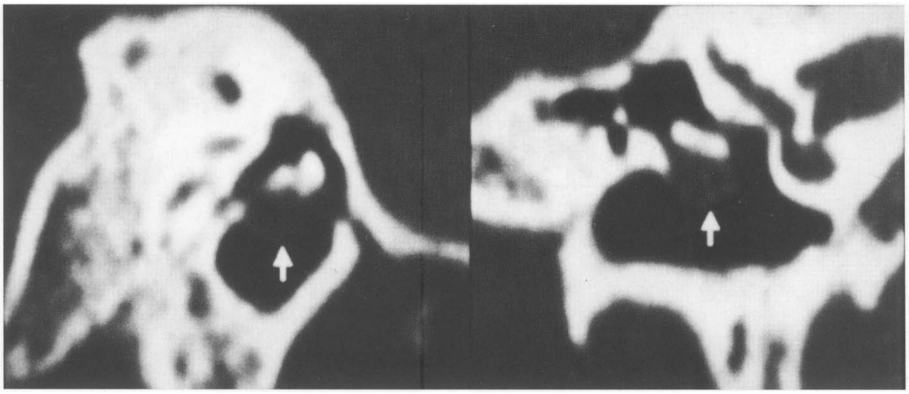

FIGURE 2 CT scans in sagittal and coronal planes of group A.

### RESULTS

### CT Findings

The shadow was localized lateral spread to the malleus and incus in three patients (group A) (Fig. 2) and localized in the entire epitympanum in 1 patient (group B) (Fig. 3), but extended from the mastoid antrum into the air cells in 24 patients (group C) (Fig. 4, Table I).

### Result of Surgery

The bottom of the retraction in the pars flaccida could not be observed by the trans-canal approach in any patient. An enlarged scutum and a cholesteatoma occupying the pars flaccida were the findings observed in all of the patients (Fig. 5).

### *Group A*

The shadow in the epitympanum showed localized lateral spread to the malleus and incus in 3 patients, and the spread of the cholesteatoma was limited to the lateral aspect of the ossicles. The pars flaccida was carefully observed by endoscopy and the retracted tympanic membrane was exfoliated under light vision (Fig. 6). Then the lateral wall of the attic surrounding the retracted tympanic membrane was pared away

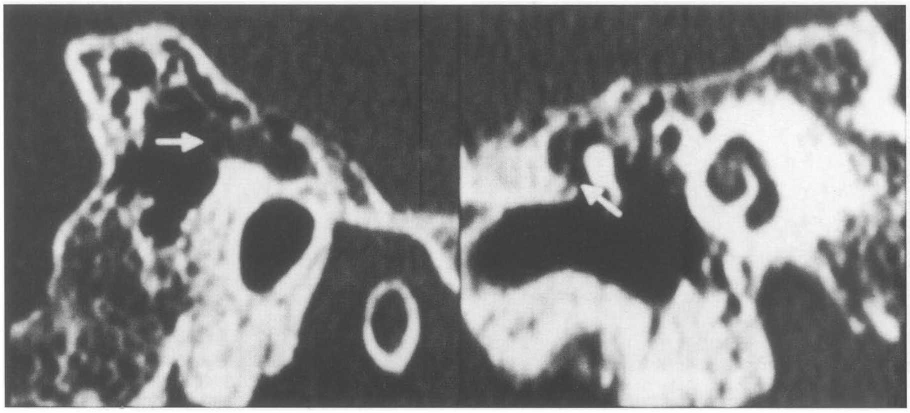

FIGURE 3 CT scans in sagittal and coronal planes of group B.

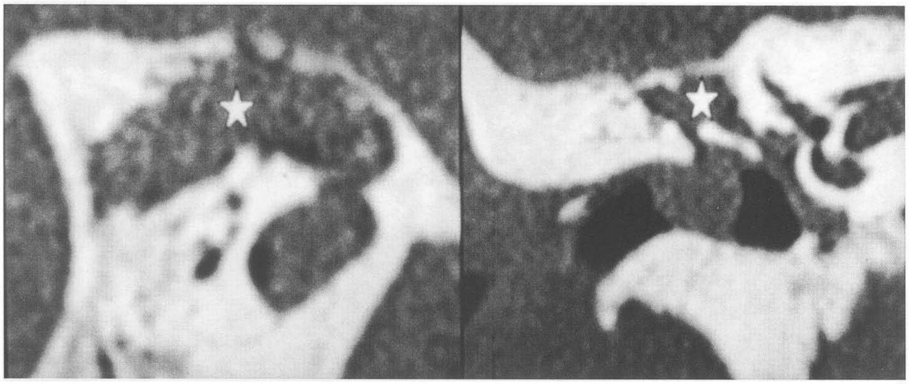

FIGURE 4 CT scans in sagittal and coronal planes of group C.

with a chisel. After freeing the retracted tympanic membrane, the cholesteatoma was completely removed under endoscopic control while observing the base of the retracted area under direct vision.

### Group B

The shadow was localized to the entire epitympanum, and a cholesteatoma that filled the entire epitympanum with destruction of the malleus head and incus body was observed in 1 patient. The retracted tympanic membrane was isolated posterio-superiorly under endoscopic control starting from the periphery of the involved area, and the tympanic membrane covering the epitympanum was completely freed (Fig. 7). The partially absorbed ossicles and an adherent cholesteatoma could be completely dissected out. After removal of the cholesteatoma, the entire epitympanum and the region extending to the aditus ad antrum could be evaluated under direct vision (Fig. 8).

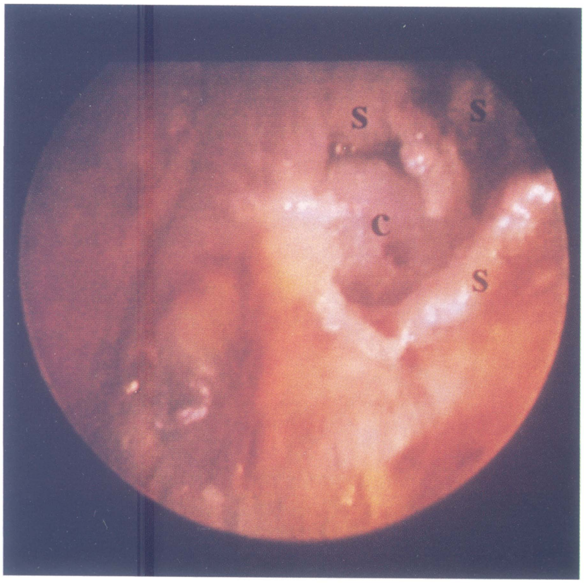

FIGURE 5 Preoperative examination of the tympanic membrane (s: an enlarged scutum, c: cholesteatoma occupying the pars flaccida).

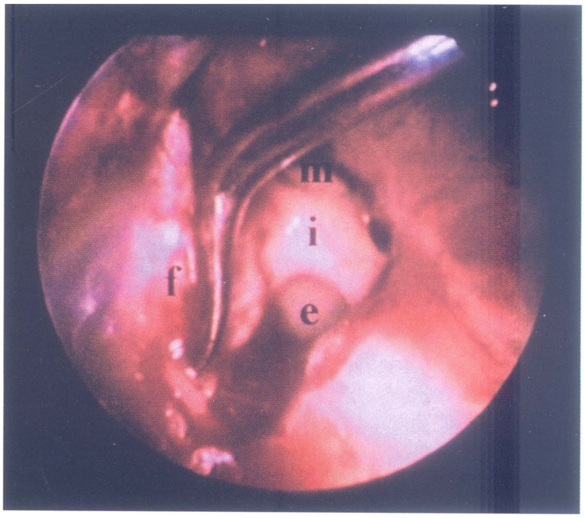

FIGURE 6 Exfoliation of the retracted tympanic membrane under endoscopic light vision (f: retracted pars flaccida, i: incus body, m: malleus head, e: epitympanum).

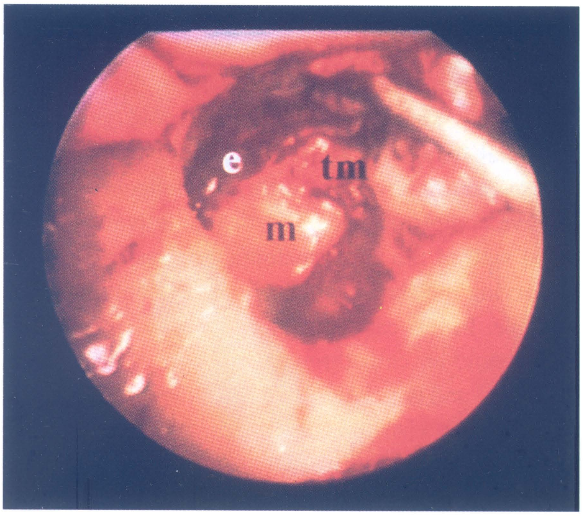

FIGURE 7 Freed tympanic membrane covering the epitympanum under endoscopic control (tm: tympanic membrane, m: malleus head, e: epitympanum).

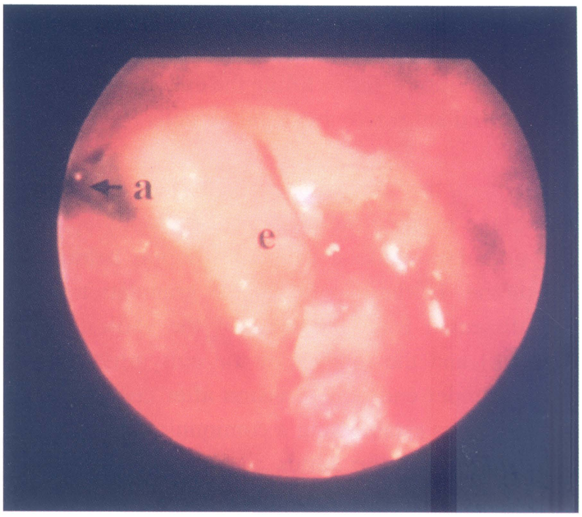

FIGURE 8 Evaluation of the entire epitympanum and the region extending to the aditus ad antrum (a: aditus ad antrum, e: epitympanum).

### Group C

The shadow filled the epitympanum, antrum and the mastoid air cells on preoperative CT scans in 24 patients, but the extent of cholesteatoma spread was classified into three groups.

(1) Slight granulation and fluid filling the epitympanum from the mastoid antrum to the distal air cells (5 out of 24 patients).

Slight granulation tissue with a large component of retained fluid was observed behind the cholesteatoma in 5 of the 24 patients. The cholesteatoma was located on the lateral aspect of the ossicles in 2 of these 5 patients. It could be resected under endoscopic control, but invasion behind the incus could not be confirmed because of the slight granulation after cholesteatoma removal. Therefore, the ossicular chain was transected, and the incus was rotated to observe the epitympanum behind the incus as far as the aditus ad antrum under direct vision (Fig. 9). After confirming the absence of cholesteatoma spread behind the incus, it was restored to its original position and the ossicular chain was reconstructed by interposing a small columella between the malleus–incus joint. As a result, good hearing was restored. In 3 of the 5 patients, the cholesteatoma had spread towards the medial wall of the epitympanum, accompanied by destruction of the malleus head and incus body. Therefore, the head of the malleus was transected and the incus was removed. After cleaning the interior of the epitympanum under endoscopic vision, the air cell cavity was cleaned by aspiration

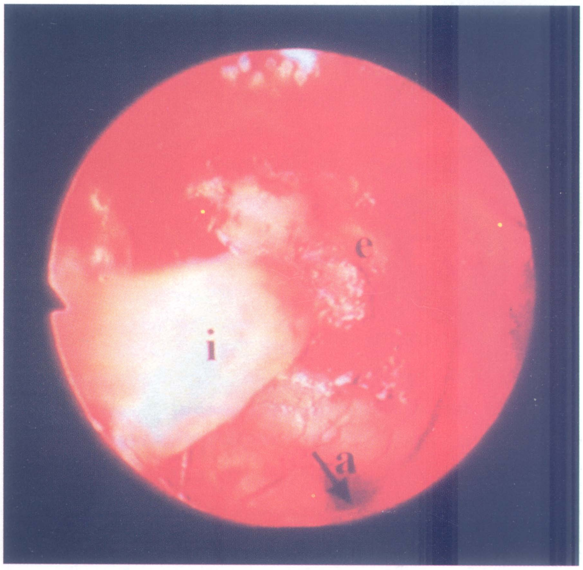

FIGURE 9 Observation of the epitympanum behind the rotated incus as far as the aditus ad antrum under direct vision (i: rotated incus, a: aditus ad antrum, e: epitympanum).

from the aditus ad antrum to its distal end under direct vision.

(2) Granulation tissue filling the epitympanum from the mastoid antrum to the distal air cells (13 out of 24 patients).

In 13 of the 24 patients, the retracted tympanic membrane reached the tegmen of the epitympanum, and granulation tissue filled the region behind the membrane. In these patients, the granulation tissue was dissected from the back of the cholesteatoma (Fig. 10) and the interior of the epitympanum was cleaned under endoscopic vision after cholesteatoma removal, thus establishing a communication from the epitympanum to the aditus ad antrum. As a result, ossiculoplasty and scutumplasty could be performed. In one of the 13 patients, a polyp was protruding into the auditory canal from the retraction pocket of the pars flaccida. In this patient, the extent of cholesteatoma spread was confirmed endoscopically and the lesion was resected together with the ossicles. After cholesteatoma removal, it was confirmed that the epitympanum had no residual cholesteatoma as far as the aditus ad antrum.

(3) Cholesteatoma involving the mastoid antrum (6 out of 24 patients).

In the remaining 6 patients, the cholesteatoma sac was dissected free from the surrounding bone of the epitympanum under endoscopic vision, but the aditus ad antrum was left with a continuous squamous epithelial remnant (Fig. 11). In these patients, transmastoid antrotomy was performed concomitantly to achieve complete removal of the cholesteatoma. The distal wall of the cholesteatoma sac was freed while preserving the surrounding mucous membrane where possible. In this case, the endoscope was passed into the mastoid air cells through an aperture made at the distal end and the cholesteatoma sac end invading the air cells was visualized (Fig. 12). It is important to confirm whether the medial part of the malleus or incus is involved after epitympanum debridement, but the medial part of the ossicles could be evaluated and the cholesteatoma could be removed under direct vision when it was observed from the antrum and epitympanum side using the endoscope that had been passed into the air cells (Fig. 13). Concomitant use of the endoscope during tympanoplasty was quite helpful when opening the mastoid antrum. It was also valuable with respect to improving the reliability of surgery, reconstruction of the ossicles, and postoperative pneumatization.

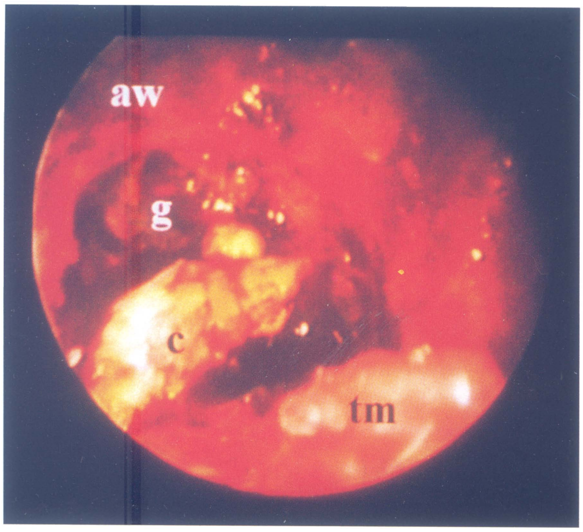

FIGURE 10 Dissection of the granulation tissue from the back of the cholesteatoma (c: cholesteatoma, g: granulation tissue, tm: tympanic membrane, aw: attic wall).

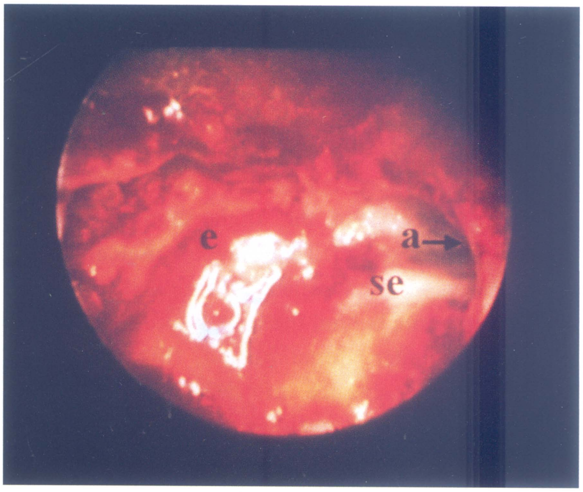

FIGURE 11 Squamous epithelial remnant towards the aditus ad antrum (se: squamous epithelium, a: aditus ad antrum, e: epitympanum).

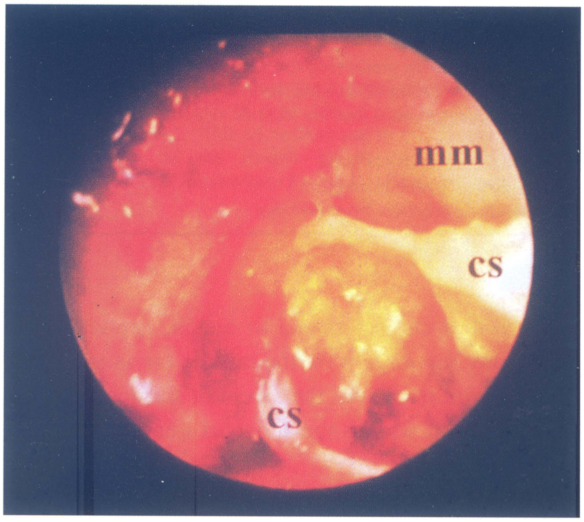

FIGURE 12 Distal wall of the cholesteatoma sac and surrounding mucous membrane (cs: cholesteatoma sac, mm: mucous membrane).

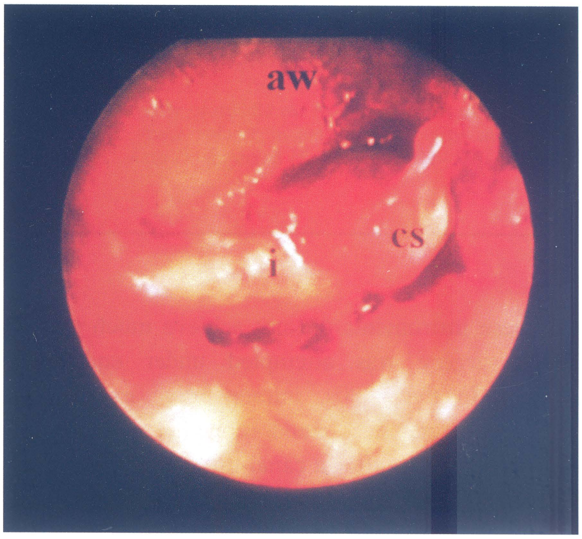

FIGURE 13 Direct vision of the medial part of attic wall, malleus and incus from the antrum and epitympanum side (aw: medial part of attic wall, i: incus, cs: cholesteatoma sac).

### Postoperative Findings

*Complication.* All patients were operated without any disadvantage of using endoscope. There were no incidents of iatrogenic injuries in this series. Ossicle destruction by endoscope was not observed in any patient.

*Postoperative pneumatization.* During the mastoidectomy, concomitant use of the endoscope was valuable with respect to preserving the mucous membrane surrounding the cavity, and it was effective to improving the postoperative pneumatization. Aeration to distal end of the mastoid process by postoperative CT findings rapidly developed in 4 of the 6 patients with mastoidectomy within 6 months after operation.

*Recurrence of cholesteatoma.* The recurrence of cholesteatoma was not observed in any patients at after 18 months of this operation.

*Hearing results.* None of the patients had worsening of sensorineural hearing loss. The pre-operative audiometric air conduction level in the 0.5–2 kHz ranged from 11–59 dB and the average level was 39.6 dB. The post-operative HL ranged from 4–48 dB and the average was 29.8 dB HL.

### DISCUSSION

When an otologist operates on the middle ear, surgery can generally be performed under direct vision with the aid of an operating microscope. However, not all parts of the middle ear can be operated on under direct vision, even when using a microscope, since there are sites that are hidden from view. Under a trans-canal approach, such sites typically include the epitympanum as well as the inferior and posterior parts of the mesotympanum. Because the ossicular chain is located in these regions, it is hard to visualize. It is almost impossible to operate on these sites using a microscope alone unless the surrounding bone is pared away. Both the surgical instruments and operative technique have been improved in many ways to cope with this problem of microscopic surgery. Jako *et al.* [1] tried to view the posterio-superior quadrant (PSQ) of the tympanic cavity using a tool with a tiny mirror at its tip. Although the PSQ could be observed, surgery was found to be impossible. Subsequently, reports appeared that strongly advocated a flexible endoscope [2] or a rigid endoscope 1.7 mm in diameter [3] for observation of the middle ear cavity, especially the PSQ,

TABLE II Advantage and disadvantage of using endoscope for ear surgery

| | |
|---|---|
| Advantage | 1. Good view with preserving the normal anatomy<br>2. Reduce the extent of surgical invasion<br>3. Gain access to sites that are hard to visualize with microscope |
| Disadvantage | 1. Narrow the approach area<br>2. Shadowed with hemorrhage<br>3. Ossicle destruction by endoscope |

but these instruments did not achieve much success when surgery was attempted under endoscopic control. In the 1980s, intraoperative examination with a 90-degree needle endoscope [4] and diagnosis of cholesteatoma after a canal-up operation [5] were reported. From around 1985, operations on the tympanic sinus were performed with the aid of an endoscope and an attached video camera [6,7]. Techniques for surgery on the middle ear under endoscopic control have been further developed along with advances in the equipment.

A rigid, thin endoscope is very effective for viewing obscure sites when performing surgery on the middle ear under an operating microscope. Once the good visibility achieved with the endoscope is appreciated, the inadequacy of the microscope becomes more obvious. The rigid endoscope is essential; for microsurgery on the middle ear because it facilitates functional reconstruction and minimizes bone removal while preserving as much of the normal anatomy as possible. Concomitant use of the endoscope is somewhat troublesome, but this approach deserves further development for exploration of the possibilities of endoscopically assisted reconstructive surgery.

There are both advantages and disadvantages of using the endoscope during middle ear surgery (Table II). However, what matters most with the trans-canal approach with endoscope is gaining access to sites that are hard to visualize directly with the microscope, in order to perform cholesteatoma surgery successfully. It is also important to restore middle ear function by efficient reconstruction after cholesteatoma removal by restoring the middle ear mucosa and middle ear structures to normal with minimal removal of bone [8]. Considering that the population is rapidly aging with a cholesteatoma to match, application of endoscopy for surgery on the middle ear is important both because it reduces the extent of surgical invasion and because it widens the scope of treatment.

### CONCLUSION

Total resection of cholesteatoma was possible by trans-canal atticotomy combined with the use of a rigid endoscope not only in 4 patients with the shadow localized in the epitympanum on preoperative CT scans but also in 18 out of 24 patients with the shadow extending from the epitympanum to the distal mastoid air cells. The mucous membrane of the epitympanum and the region from the aditus ad antrum to its distal end could be preserved intact when the cholesteatoma was removed under endoscopic control. The mastoid cavity showed a tendency for early pneumatization when the retained fluid in the antrum was adequately aspirated at the surgery. This study indicates that the trans-canal approach with endoscopic guidance is a useful technique for the treatment of attic cholesteatoma.

### *References*

[1] Jako, G. (1966) "The posterior bony ear canal wall and the antrum threshold angle in conservative middle ear surgery", *Laryngoscope* **76**, 1260–1271.
[2] Mer, S., Derbysthire, A. and Brushenko, A. (1967) "Fiberoptic endotoscopes for examining the middle ear", *Arch. Otolaryngol.* **85**, 387–393.
[3] Willemot, J., Marquet, J., Van Clooster, R., et al., (1975) "Les techniques d'endoscopie et de reproduction de l'image en otorhinolaryngologie", *Acta Otorhinolaryngol. Bel.* **26**, 299–316.

[4] Nomura, Y. (1982) "A needle otoscope (an instrument of endotoscopy of the middle ear)", *Acta Otolaryngol. (Stockh.)* **93**, 73–79.
[5] Kanzaki, J. (1983) "Postoperative aural attico-antroscopy for cholesteatoma", *ORL* **45**, 290–296.
[6] Yung, M. (1992) "Use of endoscopes in middle ear surgery", Proceedings of the Fourth International Conference on Cholesteatoma (Amsterdam: Kugler Publications), pp 577–579.
[7] Thomassin, J.M., Korchia, D. and Doris, J.M.D. (1993) "Endoscopic guided otosurgery in the prevention of residual; cholesteatomas", *Laryngoscope* **103**, 939–943.
[8] Aoki, K., Mitani, Y., Tuji, T., et al., (1999) "Relationship between severity of middle ear mucosal lesion and middle ear pneumatic space volume in patients with otitis media with effusion", *Acta Otolaryngol. (Stock.)* **119**, 562–567.

